# Regulation of T cell antitumor immune response by tumor induced metabolic stress

**DOI:** 10.15698/cst2019.01.171

**Published:** 2018-11-27

**Authors:** Fanny Chalmin, Mélanie Bruchard, Frederique Vegran, Francois Ghiringhelli

**Affiliations:** 1 Cancer Biology Research Platform, Centre Georges-François Leclerc, Dijon, France.; 2 Université de Bourgogne-Franche Comté.; 3 GIMI Genetic and Immunology Medical Institute, Dijon, France.; 4 INSERM UMR1231, Dijon, France.

**Keywords:** antitumor immmunity, metabolic stress, acidosis, hypoxia, amino acids, fatty acid, T cells

## Abstract

Adaptive T cell immune response is essential for tumor growth control. The efficacy of immune checkpoint inhibitors is regulated by intratumoral immune response. The tumor microenvironment has a major role in adaptive immune response tuning. Tumor cells generate a particular metabolic environment in comparison to other tissues. Tumors are characterized by glycolysis, hypoxia, acidosis, amino acid depletion and fatty acid metabolism modification. Such metabolic changes promote tumor growth, impair immune response and lead to resistance to therapies. This review will detail how these modifications strongly affect CD8 and CD4 T cell functions and impact immunotherapy efficacy.

## INTRODUCTION

The tumor microenvironment (TME) plays an important role in tumor progression and response to therapy. A growing number of publications show that CD8 T lymphocytes accumulation in tumor bed is a biomarker of a good clinical outcome in most cancer types [1]. Moreover, such an immune response is also a surrogate marker of chemotherapy efficacy in breast cancer setting and a biomarker of checkpoint inhibitors efficacy [2, 3]. Antitumor immunotherapy and in particular immune-checkpoint-targeting inhibitors are revolutionizing cancer therapy [4]. Checkpoint inhibitors targeting PD-1 (programmed cell death protein-1)/PD-L1 (programmed death-ligand 1) lead to a response rate in many tumor types. However, in prevalent tumor types, such as colorectal cancer, lung cancer and breast cancer, substantial responses to checkpoint blockade have only been observed in specific subsets of patients, thus suggesting that both patient selection and therapy combination may be crucial [5]. Currently two concepts evolve in parallel to predict checkpoint efficacy: the presence of mutations in tumor cells and the presence of immune infiltrate at tumor site (the concept of cold vs hot tumor). The ability of a tumor to respond to immunotherapy depends on the presence of CD8 at the tumor site. However, a CD8 infiltrate does not perfectly correlate to the checkpoint response rate, thus suggesting that in addition to the number of immune cells, functional characteristics of intratumoral infiltrating T cells must be taken into account. In addition to CD8 T cell infiltrate, many other cells influence antitumor immune response. For example, CD4 T cells are essential and different subsets are defined. Regulatory T cells (Treg) and Th2 cells have immunosuppressive functions while Th1 cells have an antitumoral effect and sustain CD8 antitumoral effects [1]. Th17 cells can have different effects depending on the tumor type but frequently promote inflammation and neoangiogenesis [6]. The myeloid component of the immune system is also important to promote antitumoral T cell immune response or to drive immunosuppression. The presence of mature myeloid dendritic cells is essential for a good immune response. Myeloid derived suppressor cells (MDSC) are an essential component of the tumor induced tolerance and the ratio of Type 2/Type 1 Tumor Associated Macrophages is important to balance immune reaction from immunosuppression versus antitumoral response [6]. The recruitment and functions of immune cells in the TME markedly vary between patients even in the same tumor type for unknown reason.

While immune response is essential to control tumor growth and to promote checkpoint inhibitor efficacy, tumor environment physical conditions may influence T cell response. Tumors are characterized by low oxygen level and hypoxia, extracellular milieu acidification, oxidative stress and glucose deprivation. In this review we will resume how these physical modifications of TME affect T cell antitumoral immune response.

## HYPOXIA

The physiological oxygen fractions called normoxia largely vary between tissues and within the same tissue [7–9]. For example, the maximum value of oxygen found in the body reaches 14% in lung alveoli but only 1% in the skin. These values have to be put in balance with the atmospheric level of oxygen of 21%, frequently used for *in vitro* experiments.

Hypoxic areas can often be found within solid tumors. The oxygen level in tumors is frequently low, below 1%, and a high level of hypoxia is often associated with poor prognosis [10]. At the cellular level, hypoxia promotes tumor cell heterogeneity, epithelial to mesenchymal transition, tumor cell stemness, migration and metastatic process, and resistance to classical cytotoxic treatments such as radiotherapy and chemotherapy [11–14]. Molecular mechanisms underlying hypoxia mainly rely on the stabilization of hypoxia inducible factors (HIF1 and 2). These transcription factors mediate the cellular response to hypoxia by regulating the expression of different genes such as proangiogenic factors like VEGF (vascular endothelial growth factor) and glycolysis related genes. Indeed, low oxygen may impair energy production via oxidative phosphorylation and requires glycolysis which is less dependent on oxygen level.

**Figure 1:**
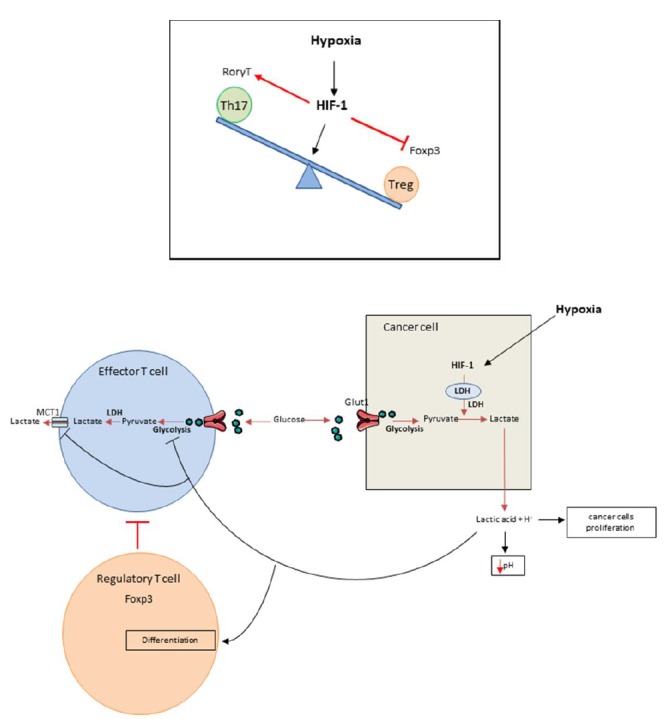
FIGURE 1. Top: Role of hypoxia in Th17/Treg balance disregulation. Hypoxia within the tumors enhances the Th17 development over the Treg development. This is due to the induction of HIF-1 that will in one side promote the expression of RORγt through its cooperation with STAT3 and in the other side bind to Foxp3 inducing its ubiquitination and degradation. **Bottom: Hypoxia promotes immunosuppression.** Cancer cells have a high glycolytic activity manifested by glucose intake through Glut1 transporter that is metabolized into pyruvate and then into lactic acid through LDH activation induced by HIF-1. The lactic acid is exported within the tumor microenvironment and induces its acidification. Effector T lymphocytes are also dependent of their glycolytic activity and must release lactate by SLC16A1; best known as MCT1. In this context of acidification, MCT1 is inhibited thus blocking the glycolysis and consequently the activation of the effector T cells. Moreover the high lactate concentration within the tumor microenvironment will promote Treg biology by inducing FoxP3 expression. In these conditions hypoxia leads to immunosuppression through the activation or inhibition of different immune cells populations.

It was recently shown that HIF-1 is able to regulate the balance between Treg and Th17 differentiation in CD4 T cells. Although TGF-β (transforming growth factor) is required for both Th17 and Treg differentiation, these cell types have opposing functions. While Th17 are pro-inflammatory cells, Tregs have an anti-inflammatory role [15–20]. Tregs master regulator is the transcriptional factor FoxP3 (forkhead box P3). In addition to TGF-β, Th17 cells require IL-6 for differentiation and expression of the transcriptional factor RORγt (RAR-related orphan receptor), the master regulator of this cell type. Hypoxia promotes accumulation of Th17 cells and decreases the number of Tregs (**Figure 1** **top**). Mechanistically, HIF-1α enhances Th17 development. HIF cooperates with STAT3 (Signal transducer and activator of transcription) to promote expression of RORγt and then cooperates with RORγt and p300 to transactivate IL-17 production. In contrast, HIF-1α blunts Treg differentiation by binding to FoxP3, promoting its ubiquitination and subsequent degradation by the proteasome [21]. While Tregs frequently promote tumor growth and mediate immunosuppression, we can hypothesize that such mechanism could promote antitumoral immune response by limiting Treg dependent immunosuppression and activating proinflammatory Th17 cells which could exert some antitumoral effects.

CD8 T cell priming under hypoxia can promote differentiation toward lytic effector cells, with increased expression of interferon gamma (IFNγ), granzym B (GZMB) and Fas ligand (FASL), but might reduce cell expansion [22–25]. Hypoxia promotes a metabolic switch from an oxidative phosphorylation metabolism toward a glycolytic metabolism [26] which promotes effector and limits memory differentiation, largely dependent on oxidative phosphorylation and fatty acid oxidation [26, 27]. Such data suggest that the use of hypoxia to generate *ex vivo* transgenic T cells or Chimeric Antigen Receptor-T cells for adoptive anticancer immunotherapy could be attractive. Moreover, it would be interesting to compare the efficacy of adoptive transfer of cells differentiated under hypoxia with increased cytotoxic effector functions and less stem cell memory properties, to the transfer of younger cells less cytotoxic with stemness capacity and better persistence and self-renewal [28–30].

Hypoxia can also affect activated memory CD8 T cells. This context is closer to tumor reality since memory cells migrate to tumor site and are then reactivated. Hypoxia prevents memory CD8 T cell expansion by decreasing both cell proliferation rate and viability, partly through apoptosis induction. Additionally, hypoxia promotes adenosine production by TME and adenosine could inhibit CD8 T cell functions. Hypoxia effect is also dependent on T cell receptor (TCR) engagement and no effect of hypoxia is observed on resting memory T cells [26]. Hypoxia could also have positive effects and enhance IL-10 production in CD8 T cells. Although IL-10 could have an immunosuppressive function, it could also sustain the development of memory CD8 T cells. In addition, hypoxia could promote CD25 and CD137 expression. CD137 is a checkpoint activator that can be targeted to reinvigorate CD8 T cells [31–33]. In some tumor models, hypoxia enhances PD-L1 expression on tumor cells and thus it might enhance the efficacy of checkpoint inhibitors targeting PD-1/PD-L1 [34].

## ACIDOSIS AND GLYCOLYSIS

Median extracellular pH in human tumors ranges between 6.9 and 7.0 (compared to 7.4 in normal tissues) [35] while intracellular tumor pH remains unaltered in tumor bed [36]. Acidification of the TME has direct protumoral functions such as angiogenesis, prometastatic effect, and resistance to radiation or cytotoxic chemotherapies and is thus associated with poor prognosis [37–62]. Acidification of TME is due to local metabolism, which favors glycolysis and lactic acid production (**Figure 1** **bottom**) [63–65]. Anaerobic glycolysis and production of lactic acid are strongly correlated with hypoxia but glycolysis could also arise in normoxic conditions. As glycolysis is energetically less efficient than oxidative phosphorylation, tumors must develop an important glycolytic flux to generate enough energy [66], this process is called the Warburg effect. Consequently lactic acid accumulates inside the TME [56], thus reducing pH. This acidification has a negative impact on T cell behavior and many studies demonstrated that low intra-tumoral pH leads to downregulation of anti-tumor immune responses [67]. *In vitro* experiments showed that at pH lower than 6.6 and similar to tumor pH [35], T cell proliferation, cytotoxicity and cytokine production are impaired [68]. This effect on T cells is rather dependent on pH than on the presence of lactate [25, 69, 70]. Interestingly, T cell function could be restored after pH neutralization [25, 69–72]. Such data underline that acidosis mostly inhibits T cell function rather than inducing T cell death [70–72]. *In vivo*, tumor-derived lactic acid also impedes anti-tumor immunity [73]. The *LDHA* (lactate dehydrogenase) gene, which codes for the LDH-2 protein, converts pyruvate into lactate. *LDHA* gene deficient tumors grow slower than control tumors in immunocompetent mice, but not in immunodeficient mice, thus demonstrating that lactate impedes immune response *in vivo*. Effector T lymphocytes are also dependent on their glycolytic activity and release lactate by SLC16A1 (best known as monocarboxylate transporter 1 MCT1). In TME, in the context of acidification, MCT1 is inhibited, thus blocking glycolysis and consequently the activation of effector T cells. In this context, we have observed reduced IFNγ and GZMB production by T cells. In humans, LDH expression in melanomas negatively correlates with T cell survival and activation [73]. On the other hand, Treg biology is promoted by high lactate concentration. FoxP3, Treg master regulator, shifts cellular metabolism from glycolysis toward oxidative phosphorylation [74]. Lactic acid inhibits T cell glycolysis leading to FoxP3 expression and promoting Treg differentiation [75–77]. Moreover, lactate uptake is required for Treg immunosuppressive effects [78]. At a mechanistic level, lactate is secreted by cancer cells via a monocarboxylate co-transporter, which induces acidification of the tumor with the release of lactate and H^+^. A high concentration of lactate and H^+^ blocks the monocarboxylate co-transporter of T cells. This blockade induces accumulation of these compounds in T cells, thus blunting glycolysis [79]. This leads to a reduction of the intracellular phosphoenolpyruvat level, a crucial glycolysis metabolite necessary for TCR mediated activation [73].

Recently it has been shown that tumor bed acidification blunts the efficacy of checkpoint inhibitors [80–82]. High LDH activity in blood is negatively correlated with the clinical outcome in melanoma patients treated with ipilimumab [83], pembrolizumab [82], or a combination of CTLA-4 and PD-1 blockade [84]. Similar results were observed with lung cancer treated with anti PD-1 [85], suggesting that combination of checkpoint inhibitors with drugs that lower tumor acidity could be interesting. Many drugs are currently tested such as glycolysis, lactate transporter and proton transporter inhibitors but also buffer therapies. Glycolysis and lactate are essential for T cell biology. Consequently, therapies targeting either glycolysis or lactate transporters are probably not ideal. In contrast, T cells are less dependent on proton transporters. Therefore, proton pump inhibitors and bicarbonate based therapies, which both can neutralize acidification, are probably better candidates to enhance immune response and to promote checkpoint inhibitors efficacy and adoptive T cell therapies.

## AMINO ACIDS

**Figure 2:**
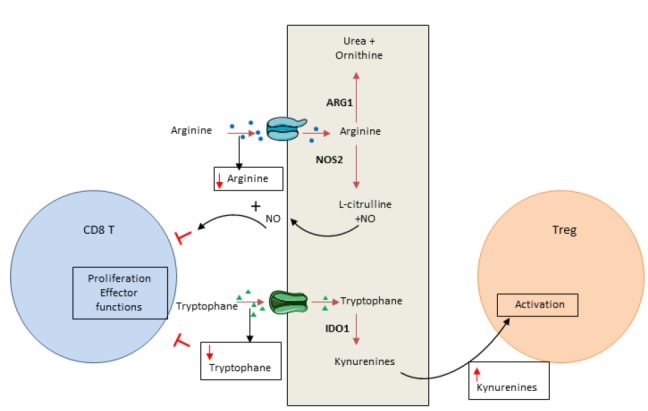
FIGURE 2: Amino acid consumption by cancer cells promotes immunosuppression. Cancer cells deplete essential amino acids for T cell activity leading to a decrease of their antitumoral function. For instance cancer cells import arginine that will be metabolized in Urea by Arginase 1 (ARG1) or in L-citruline + Nitric oxide (NO) by Nictric oxide synthase 2 (NOS2). The diminution of Arginine within the tumoral microenvironment as well as the production of NO by the cancer cells are inhibitor of T cells. The diminution of tryptophan in the tumoral microenvironment will also inhibit T cell. Moreover, the increase in kynurenine (tryptophan metabolite produced by IDO) will promote Treg cell activity.

In addition to glucose, amino acids are essential elements for energy generation in tumor cells and immune cells [86]. Cancer cells have the ability to consume a high level of amino acids, leading to T cell deprivation. Both arginine and tryptophan are essential for T cells and cannot be produced by T cell metabolism. Consequently, consumption of these amino acids by cancer cells controls the local immune response by inducing T cell metabolic stress (**Figure 2**).

Arginine can be used in oxidative phosphorylation and as a substrate for glycolysis in T cells [87]. Arginine availability favors memory T cell generation [88]. Arginine is converted by arginase or nitric oxide synthase, normally expressed in myeloid cells such as myeloid-derived suppressor cells, macrophages, dendritic cells and cancer cells. Arginase is highly expressed in many tumor types and induces T cell function inhibition via arginine deprivation [89]. Nitric oxide synthase, also frequently expressed in tumors, degrades arginine into nitric oxide. Nitric oxide could directly blunt T cell proliferation and secreting functions and promote T cell apoptosis [90].

Tryptophan is critical for several metabolic pathways and proliferation. Indoleamine-2,3-dioxygenase (IDO) 1 and 2 are key enzymes that transform tryptophan into its metabolite kynurenine. In tumors, IDO induces tryptophan deprivation and kynurenin accumulation. Tryptophan is essential for T cell biology and its depletion induces eukaryotic translation initiation factor 2 alpha kinase 4 (EIF2AK4; also known as GCN2, General Control Non-derepressible 2 kinase) activation and CD3 ζ-chain downregulation. These events reduce T cell effector functions and limit their proliferation [91, 92]. Similarly, kynurenine restrains T cell proliferation [93] and could activate arylhydrocarbon receptor, promoting the switch of CD4 T cells into Treg cells [94]. Recent data demonstrated that both cancer cells and tumor infiltrating myeloid cells could have a high level of IDO enzyme expression [95–97]. IDO is not constitutively expressed and its induction is dependent on inflammatory signal stimulation such as IFNγ [95–99]. IDO acts as a negative feedback loop of Th1 response in cancer.

Inhibition of IDO and arginase could restore T cell functions and could improve the effector T cells/Treg ratio. Multiple IDO and arginase inhibitors are currently in development, associated to adoptive T cell therapy or checkpoint inhibitors [94]. However first reports are disappointing and phase III clinical trials evaluating efficacy of combination therapies involving IDO1 inhibitors and pembrolizumab in patients with melanoma are stopped (ECHO-301/KEYNOTE-252 study).

## FATTY ACID METABOLISM

Alterations in lipid metabolism are frequently observed in cancer cells [100]. Tumor aggressiveness is linked to its capacity to store high levels of lipid and cholesterol [101–103].

Fatty acid metabolism has a role in T cell differentiation. Effector CD8 T cells use *de novo* fatty acid synthase and fatty acid uptake, whereas memory T cells degrade endogenous esterified fatty acids [104]. Endogenous fatty acid generation is essential to maintain energy level after PD-1 activation [105]. PD-1 activation impairs glucose and glutamine uptake but promotes fatty acid oxidation and utilization of endogenous lipids. Endogenous T cell lipid reserves provide energy and may be related to T cell exhaustion and T cell ability to be reactivated by checkpoint inhibitors [106]. Lipids produced by tumor cells could also have an impact on T cells by their transformation into prostaglandin by cyclooxygenase 2. Prostaglandin could then induce inflammation [107].

Concerning CD4 T cells, competition between *de novo* fatty acid synthase and exogenous uptake controls the decision between Th17 and Treg cells differentiation [108, 109]. Inhibition of acetyl-CoA carboxylase 1 and the related *de novo* fatty acid synthase restrains Th17 differentiation and promotes Treg cells. Such data suggest that in tumor tissue where fatty acids are mostly directed to tumor cells, the deficit in exogenous fatty acids promotes *de novo* fatty acid synthase and Th17 response [108]. The molecule mTOR (mammalian target of rapamycin) is essential to control Treg differentiation, function, and survival notably by its ability to control many lipid metabolism genes [110, 111].

Targeting fatty acid metabolism could be useful to improve antitumor immune response [109]. Fatty acid oxidase is required not only for memory CD8 T cell development but also for Treg cell differentiation [112], therefore its blockade limits Treg dependent immunosuppression. Similarly, fatty acid oxidase has a critical role in MDSC-mediated T cell suppressive function [113, 114]. Thus, inhibiting fatty acid metabolism may affect multiple immune populations and could have unpredictable outcomes. In contrast, fibrate which enhances fatty acid oxidase activity and enhances endogenous production of fatty acids, may enhance functions of exhausted CD8 T cells and delay tumor growth when used together with PD-1-blocking immunotherapy [115].

## CONCLUSION

TME is metabolically different from healthy tissues. Tumors are characterized by glycolysis, hypoxia, acidosis, amino acid depletion and fatty acid metabolism modifications. These modifications strongly affect CD8 T cell functions and T helper cell differentiation. Consequently, better understanding of tumor environment metabolic changes will provide key information for the development of novel therapies that improve T cell immune functions. A better knowledge of the metabolic pathways not shared between cancer and immune cells will allow the selection of drugs targeting specifically cancer or immune cells. The use of these novel drugs in combination with immunotherapies such as checkpoint inhibitors or adoptive cell transfer may open new opportunities to improve cancer treatment.

## Abbreviations:

HIF: hypoxia inducible factor
IDO: indoleamine-2,3-dioxygenase
LDH: lactate dehydrogenase
PD-1: programmed cell death 1
PD-L1: programmed death-ligand 1
ROR: RAR-related orphan receptor
TME: tumor microrenvironment
Treg: regulatory T cell
